# A harmonised, high-coverage, open dataset of solar photovoltaic installations in the UK

**DOI:** 10.1038/s41597-020-00739-0

**Published:** 2020-11-13

**Authors:** Dan Stowell, Jack Kelly, Damien Tanner, Jamie Taylor, Ethan Jones, James Geddes, Ed Chalstrey

**Affiliations:** 1 Centre for Digital Music, Queen Mary University of London, London, E1 4NS UK; 2 Open Climate Fix Ltd., London, UK; 3 The Alan Turing Institute, London, NW1 2DB UK; 4 Sheffield Solar, The University of Sheffield, Hicks Building, Hounsfield Road, Sheffield, S7 2QZ UK

**Keywords:** Energy infrastructure, Photovoltaics, Climate-change mitigation

## Abstract

Solar photovoltaic (PV) is an increasingly significant fraction of electricity generation. Efficient management, and innovations such as short-term forecasting and machine vision, demand high-resolution geographic datasets of PV installations. However, official and public sources have notable deficiencies: spatial imprecision, gaps in coverage and lack of crucial meta data, especially for small-scale solar panel installations. We present the results of a major crowd-sourcing campaign to create open geographic data for over 260,000 solar PV installations across the UK, covering an estimated 86% of the capacity in the country. We focus in particular on capturing small-scale domestic solar PV, which accounts for a significant fraction of generation but was until now very poorly documented. Our dataset suggests nameplate capacities in the UK (as of September 2020) amount to a total of 10.66 GW explicitly mapped, or 13.93 GW when missing capacities are inferred. Our method is applied to the UK but applicable worldwide, and compatible with continual updating to track the rapid growth in PV deployment.

## Abstract (summary)

**Table:** 

Measurement(s)	geographic location • power • photovoltaic system • solar power station
Technology Type(s)	digital curation • computational modeling technique
Factor Type(s)	installation geographical size
Sample Characteristic - Environment	stellar radiation • power plant
Sample Characteristic - Location	United Kingdom

Machine-accessible metadata file describing the reported data: 10.6084/m9.figshare.13050869

## Background & Summary

Renewable energy sources now contribute a significant fraction of the world’s energy mix^1,2^, and this is set to grow. However, estimates of current capacity are surprisingly uncertain: as of 2018, the world’s solar photovoltaic (PV) installed capacity was estimated by BloombergNEF as 526 GW, REN21 as 505 GW^2^, and by IRENA as 486 GW^3^. Tracking of even a single country’s installed capacity, even retrospectively, is often fraught with inaccuracies and omissions due to market fragmentation and changing regulations^4^. This is not just a question of accounting. Reliable open data about renewable power sources will enable significant additional CO2e (carbon dioxide equivalent) savings—through various means including short-term output forecasting^5,6^ [Section 1.1.1], demand forecasting^6^ [Section 1.1.1], fleet management, and capacity expansion—and thus contribute significantly to reducing the impact of the climate crisis at national and international scale^7^. “Open data” here refers to data published and licensed permissively in such a way that it can be reused by third parties for a very wide variety of purposes, without need for any direct licensing arrangements; at the national scale, open data has significant economic benefits^8–11^. In the UK, the creation of such open data was one of the key recommendations of the recent Energy Data Taskforce^7^.

In the power sector, many data sources are not open, for reasons commercial as well as historical. Even where data are publicly available, many small-scale solar PV systems, even when connected to national grids, are not officially registered, or not registered in the same detail as are large sites. The immaturity of the renewable sector also has an impact, where changes to feed-in-tariff schemes have left gaps in the national accounting.

Open data, citizen science, and machine learning methodologies offer ways to fill these gaps. In this paper we present a methodology for this as well as an open dataset of solar photovolatic (PV) power covering the UK which offers high coverage of solar generators both large and small, with rich detail of metadata and location geometries. Our dataset contains data in greater detail and for substantially more installations than any previous open dataset. The method we present is fully open, can be reused directly in other countries, and produces data in sufficient detail to support modern applications such as outturn modelling, short-term power forecasting and machine vision. We first give a brief overview of these applications, before listing existing datasets and the gap we fill with this work.

### Applications: short-term solar PV forecasting, machine vision

Solar photovoltaics (PV) is one of the most significant sources of uncertainty for many national electricity power forecasts. Since PV outturn can fluctuate rapidly in line with regional sunshine, a Transmission System Operator must mitigate against the high degree of uncertainty in PV generation availability. They typically keep natural gas generators operating at less than full capacity so they have headroom to ramp up quickly to fill a gap in PV generation. This is financially expensive and carbon-intensive. Hence, accurately forecasting solar PV power generation a few hours in advance could significantly reduce CO2 emissions and reduce the costs of balancing the grid. Rolnick *et al*.^6^ [sec. 1.1.1] identify short-term forecasting of solar PV generation (and demand) as a particularly high-leverage opportunity for tackling climate change with machine learning. High-quality forecasts, in turn, require detailed maps of the installed capacity of solar PV power generation.

Where geographic information about PV is needed, one potential source is machine vision. Datasets of aerial imagery, from satellite or plane, can be combined with machine learning detectors to enable large scale georeferenced detection of installations. Publicly open imagery data such as Sentinel can be used^12^, but are relatively low-resolution, offering no prospect of detecting smaller installations; high-resolution imagery is typically unavailable or expensive. Object detection in images can be performed with various methods, but in recent years data-driven deep learning using convolutional neural networks (CNNs) has revolutionised the domain, having excellent performance when good large training datasets are available^13^. For a given patch of aerial imagery, a CNN-based detector could be developed to make an overall yes/no decision, or to yield more detailed pixel-wise segmentation. The extent to which the more detailed segmentation is achievable is dependent on the quality of the imagery, and the available training data, e.g. the precision of ground truth geolocations as points or outlines. Irrespective of imagery source, large and well-labelled ground truth data are vital, both for training and for evaluating detectors. Enabling such work is a goal of our dataset development.

Yu *et al*.^14^ described a CNN-based method trained on over 50 cities/towns across the USA then applied to every urban area in the contiguous USA, within which they detected 1.47 million installations. They considered both residential and non-residential PV, treated as separate object types due to their different appearance in imagery. Commercial satellite imagery from Google was used (at high 15 cm resolution), and analysed using a two-stage process: first a yes/no classifier for each image, followed by an innovative semi-supervised segmenter to provide pixel-wise segmentation. The ground-truth labelling was crowdsourced from naïve users using Amazon Mechanical Turk. Note that the imagery data are commercial, and although provided publicly, they are not open data; this means that any detections derived from such imagery might have downstream licensing restrictions. The full geographic detection data from Yu *et al*.^14^ are not published, but a summary of PV installation counts per census tract (72,537 regions).

Malof *et al*.^15^ used a CNN to perform pixel-wise detection to a single US state, trained on hand-labelled data for 3 California cities. They do not state which source of “high-resolution overhead imagery” was used. The authors explore an important issue: once a detector is trained, applying it to a new geographic region is not trivial, because the region may differ either in the imagery or groundtruth characteristics. In machine learning this is referred to as “distribution shift”. The authors mitigate the issue by fine-tuning: using a small amount of hand-labelled data for the new location to adapt the detector.

Among other things, these examples illustrate the high value of good-quality groundtruth annotated data, even when machine vision detectors are available.

### PV installations: large and small

Yu *et al*.^14^ made a distinction between small-scale (residential) and large-scale solar: this distinction is important to bear in mind, for multiple reasons. Firstly, the type (residential/commercial/utility) implies differences in the size and capacity, but also the geographical siting and the grid connectivity of an installation. It also may mark a distinction in the nature and completeness of data available. For the UK, the Renewable Energy Planning Database (REPD) is a key source of open data, including details such as capacity, installation date, and approximate location, though it only covers installations of 150 kW and above and the coverage is not exhaustive. In some countries, including the UK, small systems may legally be grid connected with no registration, meaning there may be no official documentation of their existence.

Salient for automatic detection, most utility-scale PV installations have their generators organised as large, regular, ground-mounted arrays oriented due south. Domestic rooftop arrays are more opportunistically sited, often following the orientation of existing sloped roofs. Their signature in aerial imagery is thus dramatically different. Smaller installations are of course also harder to detect using automatic methods, especially if using publicly-available (low resolution) imagery datasets.

It is important to emphasise that the “long tail” of smaller PV installations should not be neglected in any analysis of a nation’s PV capacity, nor in applications such as PV forecasting. Although an individual installation may have capacity hundreds of times smaller than utility-scale systems, their large number means they form a significant fraction of the installed capacity. In the UK, around 99% of PV installations are small-scale (<50 kW), and they account for around 30% of the nation’s installed capacity^16^.

### Existing PV datasets

#### Official datasets (UK)

Our methodology is applicable across many countries. However, the focus for our paper and dataset is on the UK, and so we review existing data sources covering the UK; for many countries, at least in the global north, there will be many similarities. As of June 2020, the UK Government reports that the country has 1,037,150 PV installations^16^.

In many countries, official data derives primarily from information submitted for two purposes: securing planning permission for a PV installation, and registering a PV installation for subsidies, tax relief or feed-in tariff scheme. In the UK, the Renewable Energy Planning Database (REPD) provides a structured and public source of data for installations of 150 kW and above—hence, largely for utility-scale and some commercial/community PV schemes. Installation capacity information is available, though the capacity of an operational scheme often differs from that cited in planning. Locations are given via addresses and postcodes, and hence geolocation is approximate especially in the countryside. When an existing solar farm is later extended, this occurs in REPD as a new entry. In some cases it is unclear whether the quoted capacity relates only to the extension or to the overall site. REPD usefully includes a field giving the status of the installation, e.g. approved/under construction/operational. However this may be incomplete or slowly updated, meaning that the operational status of some sites is uncertain.

A second source of official data in the UK comes from the feed-in tariff (FiT) scheme, which was fully operational between 2010 and 2019. This scheme registered many domestic installations as well as others. The UK Office for Gas and Electricity Markets (OfGEM) published regular summaries of FiT registration data, including installed capacity. The geolocation data are approximate, simplified for data privacy purposes from full addresses to postcode district and Lower Layer Super Output Areas (LSOA), the latter being a statistical unit used to facilitate the reporting of small area statistics e.g. census. UK FiT data updates were published periodically; however the lack of a primary key for the data makes it hard to reconcile fully the available FiT data. The UK government ended the FiT scheme from April 2019, and thus newer systems will not be represented in that dataset.

A further UK open data initiative, first published in June 2020, is the Embedded Capacity Registers published by Distribution Network Operators (DNOs). This register covers systems of 1 MW and above, and hence a subset of REPD, though includes more detailed information such as topological network connection data.

#### Commercial datasets

Solar Media Ltd publish a “UK Ground-Mount Solar Completed Assets Report”^17^ which includes detailed information for all ground-mounted solar PV systems in the UK with nominal capacity over 250 kWp. The report gives precise locations, albeit often geocoded from the postal address for the land on which the system is built (e.g. the farmhouse which owns the land). The availability and quality of system information is generally of much higher quality than the publicly available reports, though the coverage is limited to only large-scale ground-mount systems. Naturally there is a large overlap with both the REPD and FiT dataset, which are difficult to cross-reference without significant manual effort. The dataset is not publicly available and must be purchased under license.

The Microgeneration Certification Scheme (MCS) is a company created at the behest of UK government to develop standards for microgeneration technologies and provide certification services for products and installers. Registering PV systems with the MCS was a precondition of qualifying for government subsidies such as the FiT and the Renewable Obligation (RO) scheme and as such the vast majority of PV systems were historically recorded by MCS. Since the closure of the FiT and RO subsidies, anecdotal evidence suggests most small scale systems continue to be registered with MCS, presumably because the vast majority of installers who continue to operate in the UK are already certified and MCS accreditation is seen as a mark of quality. As per the FiT dataset, the MCS database includes the locations, install dates and capacities of the majority of small to medium scale PV systems in the UK. The MCS database is not publicly available and at the time of writing is not available to purchase commercially.

#### Data from novel sources

A significant new source of open data is from online crowdsourcing projects. In this work we make extensive use of OpenStreetMap, which we describe later. To give another example, PVoutput.org is a worldwide crowdsourced database for PV owners to contribute generation data from their own system, including over 2000 self-reported systems in the UK. The site focuses on time-series of generated power from each system, and also registers core attributes such as capacity and location. The data are not fully open, though they have been used for some studies of generation.

Initiatives based on automatic detection have in some cases published data derived from their systems. Yu *et al*.^14^ publish estimates that are gridded (i.e. low-resolution) covering the whole contiguous USA. As with other data sources, the coverage is more reliable for large systems than small ones.

#### Gaps in existing data sources

It is common to assume that a government data source should be treated as a gold standard, especially where (as here) the data are published under well-understood open data licences. However, there are always limitations of scope and resolution, and there are always missing data. More significantly, these data sources may have inaccuracies. In the UK, one large site (Shotwick) is recorded in the REPD as 45.7 MWp, whereas its capacity in operation is 72.2 MWp. (The smaller value presumably relates to an early-stage planning proposal.) We are crucially concerned with the accuracy of geolocations: in datasets these are typically address-based and collated primarily for correspondence or ownership purposes. These will not be accurate to the standard desired for machine vision or PV forecasting, and may represent significant anomalies if the solar array is not truly represented by the location given. As we will see, the mismatch can be on the order of a kilometre.

In many data sources, the location of a solar array is given as a single point. An ideal data source would also give the extent of the array as a (multi-)polygon, especially if it is to be used for pixel-wise detection through machine learning. These are laborious to collect, especially when precise outlines are desired. Note for example that Yu *et al*.^14^ employed a workaround using weakly-labelled data since polygon outlines were not available: if there was even a partial source of polygon data, the performance of automatic detectors could be much improved.

For good PV forecasting, certain attributes of a solar installation are helpful information: generator capacity, but also orientation and tilt to model how generation will change with the Earth’s rotation and to accommodate micro-climate effects (e.g. in coastal regions). For historical analyses and validation purposes, it is also necessary to know the commissioning date of the system. Such information may be available from the planning data for large installations, but is typically unavailable for domestic and commercial PV. (Some of these can be estimated or substituted with proxies such as the number of modules or the surface area.) The UK collected some of this information for sites registered for FiT, but for data protection reasons full details are not made public.

For the present purposes, a significant gap in existing data is simply coverage of small-scale rooftop solar PV installations. Large-scale PV can be characterised from multiple sources, almost to the extent it could be considered a solved problem, but smaller installations are much less clearly characterised. A further issue is that where multiple sources could be combined, the non-trivial tasks of data reconciliation and de-duplication become important, since for example a single system could be represented once in REPD and once in FiT.

### Crowdsourcing, citizen science and OpenStreetMap

OpenStreetMap (OSM) is a global project to create crowdsourced geographic information which anyone can use for map-making, transit routing, and many other applications^18^. It centres around a single geographic database, as well as a large and heterogeneous community of contributors that negotiate and maintain the information^19^. Like Wikipedia, OSM takes a radically open approach: anyone can edit, and there is almost no centralised policing of contributions. Instead, communities of practice emerge and evolve within the project. Analogously to Wikipedia, the outcome of this radical openness is that OSM has risen to become one of the largest, most complete, and most detailed global sources of mapping data^19^. It is now a preferred data source for many commercial providers of maps, routing, and other geographically-informed services. In some parts of the world, OSM can be of equal or better quality than commercial geographic datasets—surprisingly, this can be true even in the global North in which there are many commercial mapping providers^20^. However this depends on the level of OSM community mapping effort in a locale, which is not uniform^21^.

OSM data is not constrained to any particular application domain; it includes detailed geographic mapping of power infrastructure, thanks to OSM contributors with industrial or personal interest in that information. Recently Dunnett *et al*.^22^ used a snapshot of OSM data as a global listing of solar and wind power installations, reported as a simplified dataset of geolocation points with capacity values. They also merged some official open data sources including REPD into this data, using a spatial join to connect spatially overlapping entries, and spatial clustering to aggregate solar arrays into their corresponding farms.

**Fig. 1:**
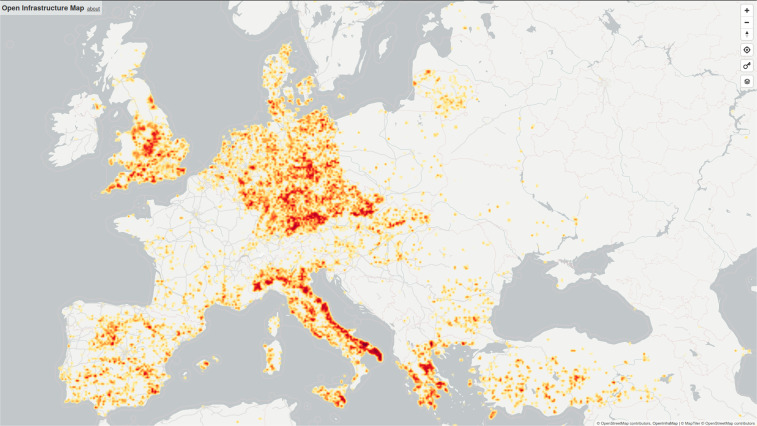
Solar PV explicitly tagged in OpenStreetMap, as of May 2020, shown as a heatmap of power capacity subtotals. Note the substantial variation between countries, often due to specific mapping initiatives in country communities: for example the relatively low density in France is unreflective of that country’s installed capacity. Image credit: Russ Garrett, openinframap.org.

Since OSM is crowdsourced, it is important to remember that sampling effort is not geographically uniform, and data consumers should be aware of this when consuming OSM data. For our particular case, note that the density of solar PV mapped in each country varies widely, according to the reality on the ground but also to local community effort and priorities in mapping (Fig. 1). To address the aforementioned issue of uneven coverage, initiatives in other domains have begun to perform coordinated region- and topic-specific efforts to add data to OSM. Most notable is “Humanitarian OSM Team” in which volunteers work together to map a crisis-affected region, mapping the basic network of buildings, roads and settlements to facilitate humanitarian relief^23^. This approach is strongly validated by practice: significant humanitarian organisations now routinely use OSM data to guide deployments^24^.

In many OSM mapping initiatives, the three main information sources are (a) aerial imagery, (b) GPS traces and (c) local information from citizens. The first two primarily help to capture large-scale aspects such as the road network, while the latter information source fills in a vast amount of additional detail. Official (e.g. governmental) data sources can be used, if openly licensed. However, a very common finding is that official data sources have quality issues of their own, often because they have been collected for a narrow purpose. Hence official data are almost never directly imported into OSM, but may be used as a source of information for manually-curated improvements.

It is important to emphasise that OpenStreetMap is not merely a database. OSM’s value lies partly in its community, a large and disparate group of people whose attention helps to maintain and update data, to negotiate evolving standards of tagging, and much more. To arrive at high-quality OSM-driven data for a particular purpose, the effective approach is to work with the OSM community in a mode of co-creation, with processes in place to extract up-to-date data as needed. This lesson has been demonstrated in humanitarian GIS applications^24^, and is the methodology used here.

## Methods

**Fig. 2:**
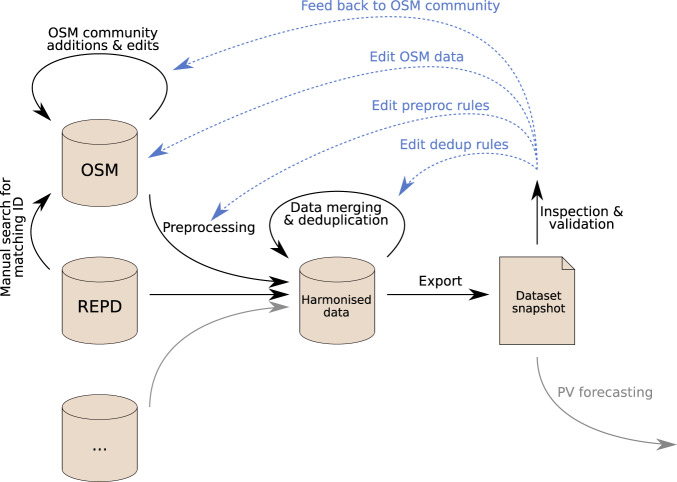
A high-level summary of our workflow. The blue arrows (upper right) show feedback mechanisms that lead to changes in data *or* in processing rules.

Our goal was to create a fully open geographic data source for solar PV within a country, suitable for applications such as machine vision and PV forecasting. To achieve that, we employed a methodology using OSM as a platform for crowdsourcing a large amount of data, followed by a pipeline of data merging, processing and validation, to produce an enriched and refined dataset (Fig. 2). We next describe the steps of this process in more detail.

### Crowdsourcing

Scoping and piloting began in early 2019. We determined that OSM’s licensing and data model could fit the task—however, fewer than 5000 PV installations had been mapped for the entire UK. We initiated discussion through the local OSM mailing list as well as social media, and interested members began to map PV installations in their local area, and also to use REPD as a guide to searching for larger farms. Solar mapping was formalised in summer 2019 when the OSM UK community took on solar PV as its “quarterly project”, focusing community effort and attention for a three-month period of collaborative work. In preparing this, we discussed which attributes of PV would be important as well as feasible to capture. This led to an agreed set of target attributes, including capacity, compass orientation, mounting location (e.g. roof or ground), and surface area and/or number of modules (either of which might serve as a proxy for capacity)^25^.

**Fig. 3:**
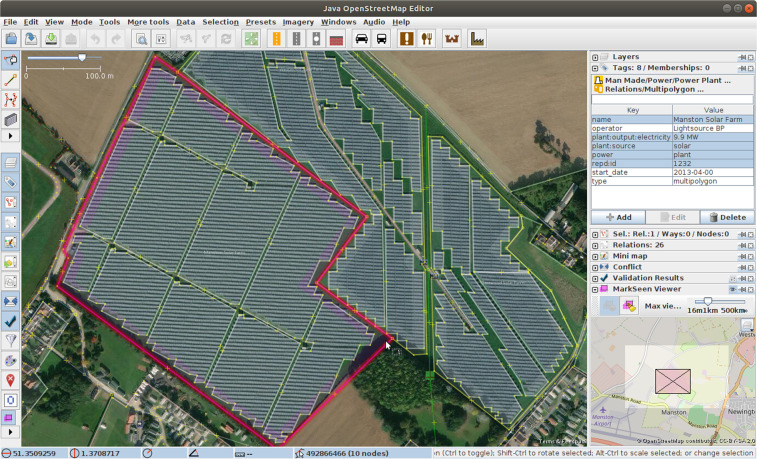
Mapping a solar farm using the OpenStreetMap ‘JOSM’ software. Solar arrays are traced using aerial imagery, and metadata added where known.

OSM UK community members took part by mapping PV installations from their local area by direct observation, but also to a large extent by searching in freely-available aerial imagery (Fig. 3). Street-level photography was also used, from members’ own collections or from openly licensed street-level photography (Mapillary).

The UK’s REPD database of large solar farms was also used by community members. The database includes approximate geolocations (addresses/postcodes), but these were not imported directly, instead used as a guide for searching. It had already been observed that these official locations related more to the registered correspondence address for the site and not the precise location of the solar array, an issue we will return to when analysing our results. Items in REPD that were mapped in OSM could be tagged with the REPD identifier, to enable direct cross-referencing. Large solar farms could be mapped in detail as (multi-)polygons. Smaller items such as rooftop PV might be mapped as polygons or simple points, if a precise extent was hard to obtain from imagery.

**Fig. 4:**
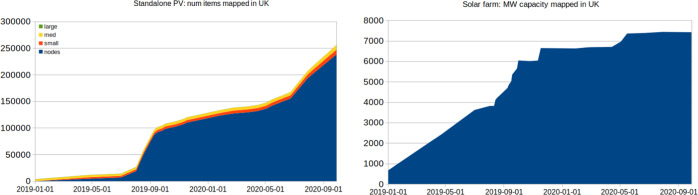
Progress of the crowdsourcing over a span of approx 18 months, showing (left) the number of standalone PV installations and (right) the total capacity of solar farms mapped.

The community crowdsourcing initiative was extremely successful. As of September 2020, over 260,000 separate UK PV objects were found, of which over 255,000 were stand-alone installations, 1067 solar farms (i.e. larger areas tagged as “power plant”), and the remainder were details of subcomponents within farms. Data were contributed by 343 distinct community members around the UK, following a strongly skewed ‘long-tail’ distribution of contributions: there was a median of 3 PV installations per person, with 95% of the installations added by the 11 most active contributors. The fastest rate of accumulation took pace during the OSM UK community “quarterly project” in summer 2019; a further high rate of accumulation took place in spring–summer 2020, due to mapping being an activity available to community members while at home during the Covid-19 UK lockdown, and facilitated by a release of updated Bing imagery for mapping (Fig. 4). The large majority of items were mapped as ‘nodes’, i.e. single points, which are very quick to add in most editing interfaces.

### Preprocessing

Pre-processing involved both manual edits (to fix individual typos in the uncontrolled OSM source data) and automated edits (e.g. standardisation of units such as kW/MW) to each of the input datasets, to bring them into a controlled format within a PostgreSQL database with type constraints for columns^26^. Since the data schema of OSM is very open and uncontrolled, users gave a wide range of tags and values. This included typos—which we handled either via preprocessing rules, by editing the data source directly, or feeding back to the individual mapper (Fig. 2)—as well as unexpected but meaningful values. For example, for an East-facing installation, the value of this tag might be given as 90, E, or EAST; cases such as these were handled in preprocessing (cardinal directions converted to degrees). There could also be meaningful values which had not been planned for, including FLAT or heliostat; for the present study, these cases were left as n/a for orientation.

We also applied preprocessing to the official REPD dataset, using the September 2019 snapshot for development, and for our released data using the more recent July 2020 snapshot. The total number of listings for UK solar farms in the development data was 2063. The number listed as “Operational” was 1024, but the total number of PV farm entries that are not labelled as being discontinued (i.e. withdrawn, refused, abandoned, or expired) was 1465. We were aware of multiple solar farms for which the official data did not yet show they were operational. Hence we used the larger set of 1465 REPD entries for matching. Note however that this could lead to an overestimate since the data might include some decommissioned PV farms that were not yet flagged as such.

### Data merging and deduplication, and area-adaptive clustering

Having the initial preprocessed datasets, the main stages of processing were to merge them together, which involves matching items across datasets, but also deduplication by matching items within each dataset. Within-dataset duplication can arise for many reasons: within OSM data there were in many cases multiple entries corresponding to geographically separated parts of a PV system; sometimes these were manually tagged as being related, but often were not explicitly linked; within REPD data there were many cases in which a PV farm would have one entry corresponding to its original planning proposal, and a separate entry for a later extension. Hence it was important to apply methods not only for reconciling items across datasets, but also to cluster and combine items within a dataset. The core of our matching across and within datasets was spatial clustering.

Dunnett *et al*. applied DBSCAN to solar farm data, a widely-used clustering algorithm based on first connecting items that lie within a fixed radius of each other, and then pursuing transitive links to agglomerate small clusters into larger ones^22^. They evaluated a variety of distance thresholds for the radius-based clustering, finding no clear cutoff but selecting 400 m. For the present work, we used our own clustering implementation, likewise using a distance threshold and then pursuing the closure of the transitive links, but with a slightly stricter threshold of 300 m.

This standard radius-based clustering was used during dataset development, including most rounds of data validation that will be reported below. However, validation identified an important limitation of radius-based clustering for geographic data such as ours, having geographical objects of widely varying sizes. The radius threshold of 300 m, though stricter than the 400 m threshold selected by Dunnett *et al*.^22^, was appropriate for larger installations but could often inappropriately group many smaller installations together. Hence in a second iteration we introduced an *area-adaptive clustering*, in which the clustering threshold is selected dynamically based on the geographical area of the two objects being compared:

1 $${r}_{A,B}=2.\sqrt{{\rm{\max }}({a}_{A},{a}_{B})}$$

where *r*_*A,B*_ is the radius to use in deciding whether to connect objects *A* and *B*, and *a*_*X*_ is the surface area of an object *X*. We clipped *r*_*A,B*_ to range limits [10 m, 1500 m] to avoid extreme values. Where an object’s capacity was not known but not its surface area, for clustering purposes only we estimated the area heuristically using a relationship of 50 *W*/*m*^2^ (see later for an empirical check of this relationship). This is much lower than the typical power density of a PV panel per unit area, to allow for the fact that in OSM polygons generally encompass the space between PV arrays as well as the panels themselves.

We applied spatial clustering to the OSM data to link proximal items together. We linked REPD data items together based on exact location matches, or close location matches with very close name matches: after stripping phrases such as “Extension” or “Resubmission” from the listed names, we selected matches for which the trigram string similarity of the names was greater than 20%.

We then merged the datasets together, using metadata as well as location matching, using a sequence of data matching rules. First we paired OSM items with tagged REPD identifiers with their corresponding REPD entries, using spatial proximity to resolve ties and ambiguities. We then linked together any un-paired OSM and REPD solar farms that were within 700 m of one another. This threshold was chosen empirically; it is higher than the aforementioned thresholds because for REPD linking we only needed to consider large solar farm units, and in this step there was less need for fine-grained disambiguation.

Some entries in the official REPD dataset corresponded not to individual solar farms, but instead to delocalised neighbourhood schemes, potentially covering a much larger area than a single farm. None of the available datasets gave a clear indication when to cluster individual domestic installations under such schemes. We handled these “schemes” as a special case with a 5 km search radius, and made no assumption that any clustering for these schemes could be simplified to a single object. Since these items are spatially disaggregated, and further they were unlikely to pool their generation into a single inverter but each household to supply the grid separately, for these schemes we took the official REPD capacity value and portioned it equally across the matched items in OSM.

### Data validation

To perform a formal validation of the data produced by our method, we used manual inspection of visualised data, using internal sense-checking as well as external comparison to a commercial dataset produced by Sheffield Solar (henceforth the *SS* dataset). The SS dataset had previously been produced, using data from REPD and commercial sources (SolarMedia) and then manually grouped and deduplicated, as part of ongoing work between Sheffield Solar and the UK Transmission System Operator, National Grid ESO. The SS dataset cannot be published as open data but was used to inspect and validate the open data solar datasets, here treated as a ground truth because it has certain advantages over the fully-open datasets: additional metadata (such as installation date) for many sites, and a high level of manual validation already performed.

To perform efficient validation, we created a user interface using Python Flask, which visualised one or more of the datasets and their correspondences, overlaid on satellite imagery. The interface also visualised various metadata of PV installations in question, for example to compare names and capacities against that in the original REPD source data. It enabled a user to label entries as correct/incorrect and also to indicate the nature of errors, such as incorrect cross-dataset matches or over-/under-greedy clustering. We have published the Flask interface openly online^27^.

We validated our dataset for internal consistency—for example, whether groupings were meaningful, or whether the indicated capacities appeared sensible given the object size—as well as external consistency with the SS dataset. Since alternative clustering of UK PV were available from^22^ (with partial overlap in the source data), we also inspected correspondences between our dataset and theirs (henceforth the *SD* dataset). Differences in grouping or matching were visualised in the interface, and manually inspected for correctness.

### Feedback processes

Our goal was to produce not just a data snapshot, but also a workflow for successful large-scale mapping of PV data. Hence we used a wide range of feedback processes, impacting on our own pipeline but also on the OSM community and data (Fig. 2).

Within our own pipeline, we added and refined various preprocessing rules (e.g. to fix common typing errors) as well as deduplication rules. This included the handling of delocalised neighbourhood schemes, described above.

In some cases it was appropriate to edit the OSM source data directly. Although large automated edits lie outside the OSM community philosophy, various manual edits could be made directly. More often, we would feed back to the OSM community, either in general or to a specific volunteer editor. General feedback included requests such as the types of metadata of interest (roof/ground mounting, compass orientation). Per-person editor feedback included queries of anomalous cases such as an installation tagged as 2 W—it had been intended as 2 kW—or a PV installation that had been accidentally mapped as occurring on a road rather than one of the nearby buildings. Such errors occur in all large-scale data collection initiatives. We highlight here that our feedback processes were intended not only to improve our own data curation, but also to guide the more general processes of OSM community mapping.

## Data Records

Our harmonised dataset is published in two formats for widest possible compatibility, considering the potential use of data by specialists in power, GIS, data science and machine learning. Firstly we exported our data to CSV, giving attributes for each PV installation as well as its location as a simple longitude-latitude centroid. All coordinates are given according to the WGS84 (GPS) coordinate reference system (CRS). To represent geographic structure in more detail we exported GeoJSON. The geometries originated largely from OSM, though for entries without OSM objects their location was represented as a single geolocation point derived from address data. The GeoJSON has a slightly lower number of entries (265,406) than the CSV (265,417) because a small number of complex entries are represented as two CSV rows but merged into single GeoJSON objects. Clustering is indicated via a column listing an (arbitrary) cluster ID for each installation; each installation is given as a separate record, so that cluster membership can be used or ignored as needed (Table 1).

**Table 1:** Number of installations and clusters in our data. These figures are calculated from the numbers of unique identifiers.

Data source	Number of installations	Number of clusters
OpenStreetMap	264,641	256,197
REPD	1405	1326
Harmonised	265,417	256,600

The dataset is freely available online via Zenodo^28^.

## Technical Validation

### Manual validation

Our validation inspected both the internal coherence of the dataset, as well as comparing it against the more-detailed private SS dataset, and the previously-published SD dataset^22^. We first report the outcome using fixed-radius clustering.

A systematic issue identified was that clustering was in many cases overly greedy. Of the 321 cluster group comparisons made, only 38 (12%) were considered fully correct; 275 (86%) were overly greedy, while only 2 were judged not greedy enough. The remaining 6 contained the correct number of objects but in the wrong combination. Often, objects that had been inappropriately combined were groups of domestic or modest commercial installations, i.e. unconnected installations geographically close to each other at a neighbourhood scale.

A second general finding, from the 988 OSM objects explicitly tagged with REPD identifiers, is that the official geolocations were very often different from the geolocation mapped from imagery or local knowledge (see later for a notable example). 251 such OSM items were over 500 m away from their corresponding REPD item; 61 over 1 km away; 31 over 2 km away; 1 over 4 km away. In most of these cases the OSM geolocation was the correct one, the mismatch typically attributable to the difference between postal address and precise location.

From the 1465 REPD entries, our procedure matched 1084 of them together with OSM objects, many more than the 487 matched in the previous SD dataset. The vast majority of the OSM-REPD associations were validated to be correct, with 22 incorrect. Where SD had made REPD matches, these were largely in accordance with our matches, with only 36 alternate matchings. We inspected these 36 as well as matchings which did not correspond to the SS data. SD had a very similar number of incorrect matches (21) as our data; 12 systems were mismatched by both.

In both these automatically-matched datasets, the main issue seen within these small sets of errors was the erroneous matching of a REPD entry to an OSM entry (as opposed to grouping errors). Mismatching was sometimes evident from the association of large capacities (5 MW) with small domestic systems. More definitively, the manually-matched SS dataset listed installation dates for many systems (not present in most OSM data), and these dates made some erroneous matches very clear due to mismatching year-of-installation values.

To produce the finalised dataset and improve on the over-clustering effect, we updated our procedure with the area-adaptive clustering described above. We then performed a second round of validation on the 826 clusters associated with REPD entries, and found that the area-adaptive clustering was a great improvement: the proportion of clusters validated as fully correct (in both clustering and REPD-matching) was 88%. Of the 97 imperfect matches, only 3 involved overly-greedy clustering, and 35 insufficiently greedy. Other issues noted were: incomplete geographic extent (26 cases), mismatched system (21 cases).

Automatic clustering of geographical objects such as PV installations is far from perfect. In our manual validation we found that the 300 m distance threshold we initially used (and hence also the 400 m threshold of^22^) led to overly aggressive clustering. A fixed radius is inadvisable because of the wide variety of PV installation shapes and sizes. We introduced a size-dependent clustering radius. In our data we publish all individual items, along with cluster IDs, so that downstream data users can use or ignore the clustering output as required.

### Empirical spatial clumping

Putting aside automatic clustering, in the course of manual validation we repeatedly observed the empirical spatial “clumping” of PV installations themselves. The distribution of PV capacity is far from uniform, and this is not predictable merely from fixed geographic aspects (latitude, landscape). It has been observed elsewhere that PV installations tend to clump together, for many underlying reasons. Local government incentives/regulations and patterns of home ownership influence a region’s rate of domestic installations. A subtle but important effect is ‘contagion’ of influence, in which an individual solar PV site can influence local neighbours to adopt the technology as well^29^. This is an additional source of the clumping of solar installations, not directly predictable from geographic features.

However it is important to remember that in citizen science, sampling effort is rarely uniform. Since many contributions in OSM come from citizens who document their own locale, the extent of mapping in OSM is driven in part by demographics: not just the number of citizens in an area, but other factors including their affluence^21^. It is also driven by the overlapping patchwork of mapping initiatives with varying regional and topical coverage^23^.

### Metadata attributes

**Fig. 5:**
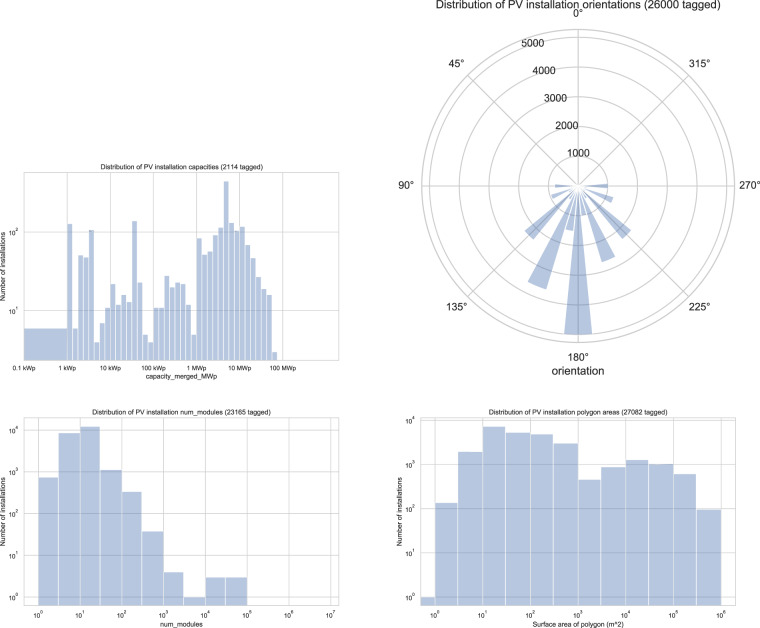
Histograms of tagged metadata in UK OSM solar panel items.

Metadata such as capacity, size and orientation of solar PV installations are present in varying amounts in our dataset (Table 2), and the distributions of their values are in line with expectations (Fig. 5). Tagged orientations are heavily weighted towards south-facing, as expected for the northern hemisphere, and show a quantisation effect with values given preferentially as one of the “16-wind” compass directions (Fig. 5(b)). This quantisation is a consequence of the tagging guidelines agreed by the community during crowdsourcing. Of the 248,391 entries tagged with location, the overwhelming majority (over 245,000) are manually tagged as “roof”. This reflects the long tail of small roof-mounted PV, and also gives a clear metadata indicator of the distinction between rooftop and larger-scale PV.

**Table 2:** Abundance of the main metadata tags in our dataset.

Tag	Count	Frequency (%)
Located (roof/ground/etc)	248,391	93.6
Surface area	27,082	10.2
Orientation	26,000	9.8
Number of modules	23,165	8.7
Capacity	2114	0.8

**Fig. 6:**
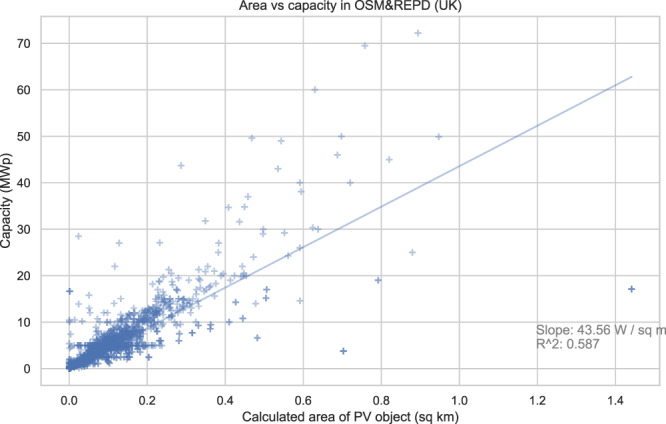
Correlation of polygon area against tagged capacity, for those OSM items which have both attributes present. Here, all categories of PV item are analysed together; we also performed separate analysis for small and for large systems, and found the relationship sufficiently similar to pool the data. Note that polygon area may cover the whole extent of a solar farm or installation, not just the PV panel surface.

To investigate the relationship between the surface area of a polygon in our dataset and the capacity of the installation it represents, we performed linear regressions. We investigated small/medium/large installations separately, finding the relationship sufficiently consistent to report it for the overall pooled data. We found a strong correlation at a level of around 44 *W*/*m*^2^) (Fig. 6), close to the known heuristic of 50 *W*/*m*^2^) used in industry. This is unrealistically low for the capacity of current PV modules— however, the polygons for many installations often encompass the entire site, including gaps between modules, and so a lower value is expected. Also note that the polygon size is judged vertically from aerial imagery, which will lead to a reduced apparent size of sloping objects due to foreshortening. The strength of this correlation despite these variability issues in mapped polygons suggests this can indeed be a useful heuristic for the capacity of PV installations if otherwise unknown. However, other factors not tagged in our dataset must also be strong predictors, such as the composition of the PV cells.

Dunnett *et al*.^22^ likewise explored the use of OSM surface area as a predictor of solar PV capacity (obtaining an *R*^2^ of 0.70). As groundtruth they used REPD for the UK, pooled with other sources covering other countries. Note that they matched far fewer REPD items to OSM objects than we did, hence the pairing of groundtruth values was different.

We also investigated the correlation between the tagged capacity and number of modules in an installation. However, there were fewer than one hundred installations tagged with both values. In addition, from notes added by mappers we know that many of these capacity values were in fact estimated from the number of modules. Thus there is no firm basis on which to use the number of modules as a data-driven heuristic for capacity here.

### Capacity totals and subtotals

**Fig. 7:**
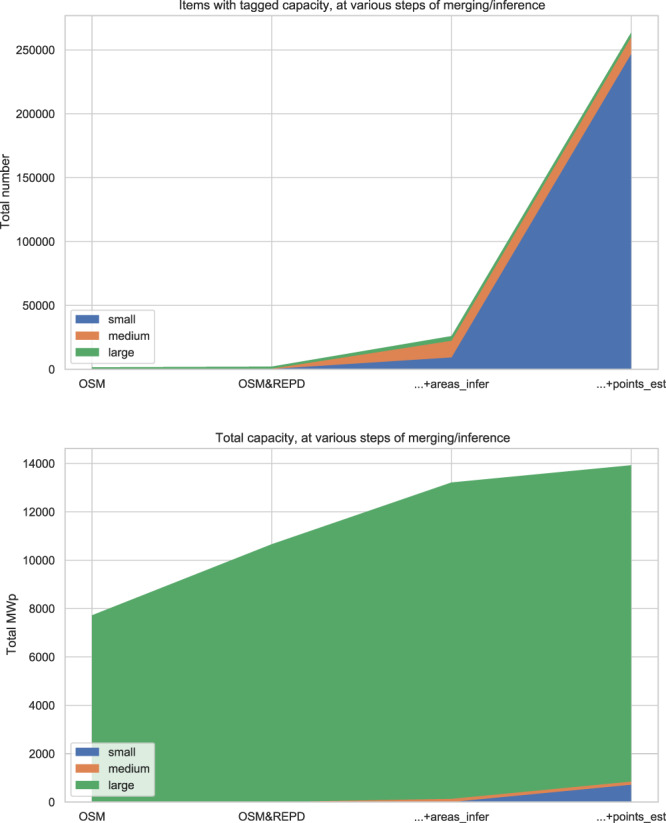
Total number of items with nonzero capacity (upper), and total capacity (lower), at various stages of merging/inference. On the left we start with OSM metadata, then merge in REPD capacities to fill in missing values. For items with surface area but unknown capacity, we then perform inference using the relationship shown in Fig. 6. Finally we make a heuristic constant estimate for the remainder of single-point items.

To make an estimate of the total capacity represented in our dataset, we consider both explicitly-tagged and inferred capacities, since there is a very large “long tail” of installations without explicitly-tagged capacity, predominantly in the small-scale part of the sector (Fig. 7). Using explicit values only, we find a total of 7.72 GW in OSM and 9.81 GW in REPD, and after merging and deduplication these lead to a total of 10.66 GW for the UK. From both datasets these explicit values are only given for around 1500 sites. The heuristic based on surface area (discussed above) allows us to estimate capacity for over 20,000 additional installations, leaving over 200,000 small installations mapped as points, for which only a naïve point estimate is possible (we use 3 kW). Including both these heuristics we arrive at 13.93 GW mapped. The UK Government estimated the UK’s PV capacity as 13.4 GW at the beginning of 2020; this may be an under- or overestimate, since it is acknowledged to omit some small/medium scale capacity which is not registered, as well as some capacity which may already have been decommissioned^30^. On the assumption (from government data) that there are in fact around 1 million installations, and that the installations as yet unmapped in our data are all small-scale, we might estimate the total UK capacity reflected here at over 16 GW, which is substantially higher than government estimates. This estimate has many uncertainties, but will be refined in future as the OSM-based data gains in richness.

**Fig. 8:**
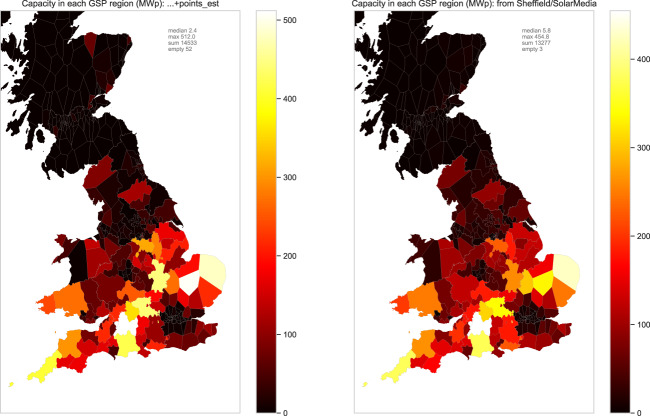
Total capacity estimate in each GSP region. Left hand: our estimates, including inferred capacities. Right hand: estimates by Sheffield Solar from commercial SS dataset.

To inspect the geographic distribution of data, and in particular the question of geographic bias due to volunteer effort, we here consider two different aggregation scales. First we group by *grid supply point* (GSP) region. For the UK National Grid, GSPs are important nodes in the graph of power connectivity, at which the transmission system is connected to the distribution system. Each GSP can be identified with a geographic region it serves^31^; we group our data according to those 329 regions (Fig. 8, left). At a finer scale, we can also group our data by *lower-layer super output area* (LSOA), which is a standard statistical partitioning of the UK into units of approximate population size 1500. These areas do not have any direct relationship with the topology of the electrical grid, but offer a finer resolution of 34,753 LSOAs.

**Fig. 9:**
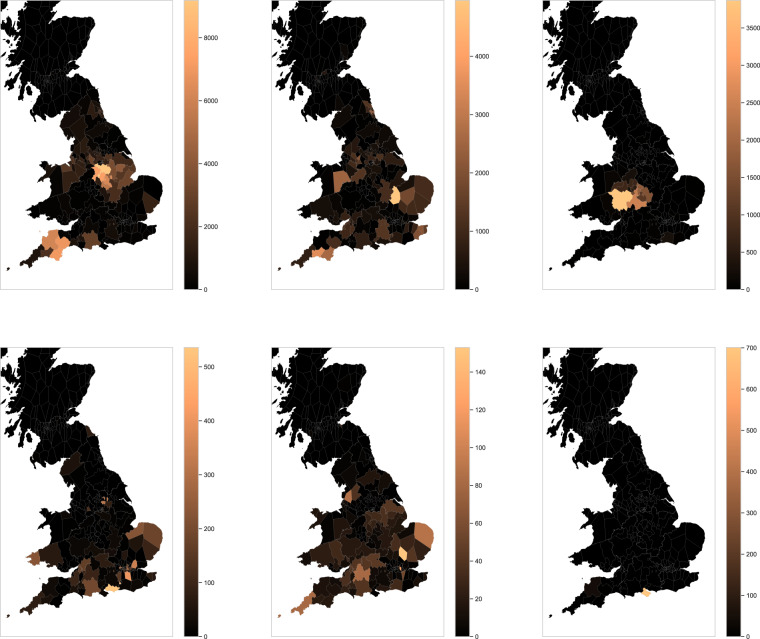
Number of items mapped in each GSP region, by each of six of the most active contributors. (Note that the overall scaling is different in each subfigure).

**Fig. 10:**
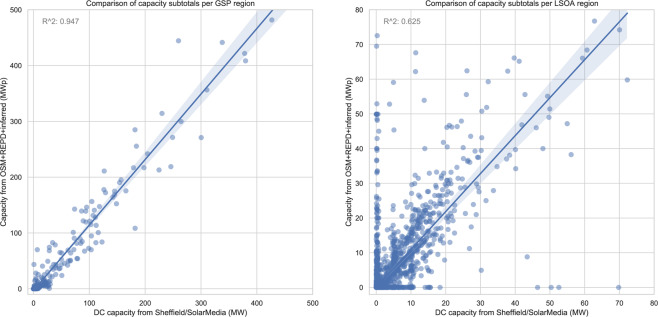
Scatter plots illustrating the correlation of capacity subtotals, in Sheffield Solar’s method (x-axis) versus our method (y-axis). We plot subtotals for GSP regions (left plot), and for LSOAs (right plot). For details of these see text.

**Fig. 11:**
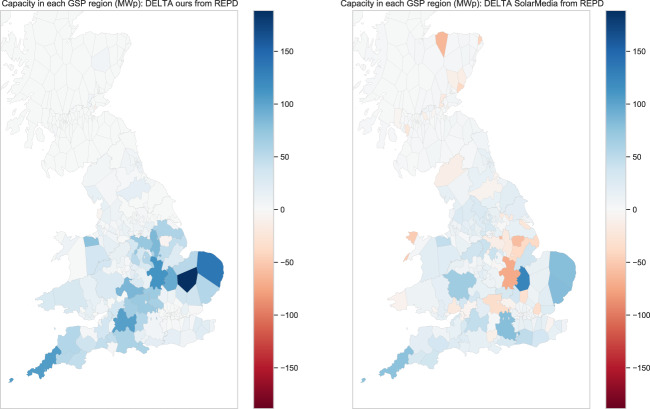
Difference plots to show the capacity data in excess of the capacities that can be obtained from the raw REPD data: ours (left plot); Solar Media (right plot).

As discussed, the spatial extent of community activity was not uniform, and this may have created biases in the data which could impact on data consumers. Inspecting the activity of the most active mappers, we see that even the most active users did indeed confined their activity to a particular UK region, while others were more widely spread (Fig. 9). Also, some focussed on small installations, while others focussed on adding and validating the data for the larger solar farm installations. Thus an individual’s impact on the logged capacity may be small or large, diffuse or concentrated (Fig. 9). To measure the extent to which such biases might affect capacity estimates, we compared our capacity subtotals against those derived from the private SS dataset (Fig. 8, right). The important assumption here is that the SS dataset has a more consistent geographic sampling; it can also be assumed to be more accurate in some ways given the more controlled manual validation procedures used in its production. Our data yields a higher estimate than SS, reflected in the above-unity correlations (Fig. 10). Both datasets contain substantial PV not represented in REPD (Fig. 11). Relative to REPD, the SS dataset coverage appears more uniform, while our dataset does indeed reflect the geographical focus of the community (compare Fig. 11 with Fig. 9). However the correlation of subtotals per GSP region is extremely strong between the datasets (*R*^2^ = 0.947; Fig. 10). At the finer scale of LSOAs, the correlation is also clear but with notable discrepancies due to geolocation differences (*R*^2^ = 0.625; Fig. 10).

**Fig. 12:**
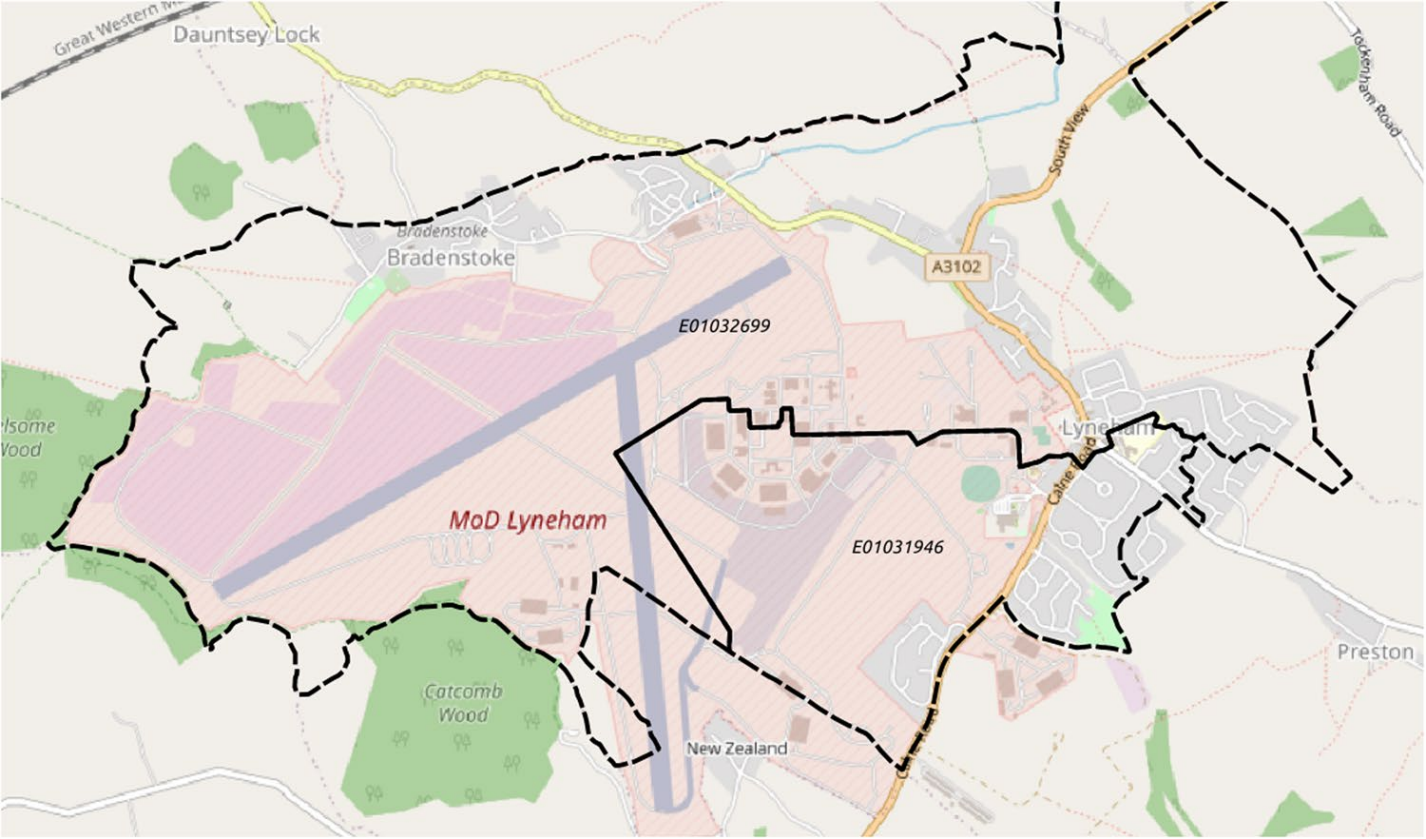
MOD Lyneham is one of the larger installations in the UK. In official data, the geolocation is given as the site entrance (grey box in lower part of LSOA E01031946); in our data, it is given as the location of the solar array itself (pink polygons on left, LSOA 01032699), well over 1 km separated. (Image basemap credit: OpenStreetMap).

A clear illustration of geospatial accuracy occurs in the case of the 69 MW installation at MOD Lyneham: using the official REPD data, its location geocodes to the site entrance, which is over 1 km separated from the solar array itself (Fig. 12). This difference is sufficient to “move” the 69 MW from one LSOA area to another (Fig. 10). Both types of geolocation are meaningful and have uses. However, for machine vision and PV forecasting the location of the array is the important information, and good geolocation is needed for good performance. Hence this and similar cases justify attention to detail when curating geolocation data. We thus recommend our dataset specifically for its high spatial precision, good coverage, and for being open and continually updated. This comes at a cost of mildly biased geographic sampling, whose effect on capacity estimates is small, but should not be neglected.

All datasets, including the ones discussed in this work, are in flux, and so continual update is a necessary attitude. Our dataset provides unprecedented coverage and geographical detail, but is incomplete. We look forward to extending coverage and metadata attributes in future work.

## Usage Notes

Our source code includes analyse_exported.py, a code example of analysing the exported data to produce most of the figures within this document, and serves also as an example of how to use the data with Python and GeoPandas. Further usage notes are given in the readme^26^.

Each record in the dataset contains cluster identifiers which can optionally be used; the cluster identifiers should be treated as opaque values.

Users may wish to aggregate power capacity across regions; we have endeavoured to ensure that power capacities are not duplicated across records, so that simple summing gives acceptable estimates (Fig. 8).

Downstream applications must be tolerant of missing data; for example distributions such as those in Fig. 5 are not presumed to be unbiased reflections of the true data distributions in the UK.

## Data Availability

Source code for producing our dataset is freely available online^26^. Requirements are Python 3.7 or later, as well as PostgreSQL with PostGIS extensions (we used PostgreSQL 10.12).
